# CACONET: a novel classification framework for microbial correlation networks

**DOI:** 10.1093/bioinformatics/btab879

**Published:** 2022-01-04

**Authors:** Yuanwei Xu, Katrina Nash, Animesh Acharjee, Georgios V Gkoutos

**Affiliations:** Institute of Cancer and Genomic Sciences, College of Medical and Dental Sciences, University of Birmingham, Birmingham B15 2TT, UK; NIHR Surgical Reconstruction and Microbiology Research Centre, Birmingham B15 2TT, UK; Institute of Translational Medicine, University Hospitals Birmingham NHS Foundation Trust, Birmingham B15 2TT, UK; MRC Health Data Research UK (HDR), Midlands Site B15 2TT, UK; Institute of Cancer and Genomic Sciences, College of Medical and Dental Sciences, University of Birmingham, Birmingham B15 2TT, UK; Institute of Cancer and Genomic Sciences, College of Medical and Dental Sciences, University of Birmingham, Birmingham B15 2TT, UK; NIHR Surgical Reconstruction and Microbiology Research Centre, Birmingham B15 2TT, UK; Institute of Translational Medicine, University Hospitals Birmingham NHS Foundation Trust, Birmingham B15 2TT, UK; MRC Health Data Research UK (HDR), Midlands Site B15 2TT, UK; Institute of Cancer and Genomic Sciences, College of Medical and Dental Sciences, University of Birmingham, Birmingham B15 2TT, UK; NIHR Surgical Reconstruction and Microbiology Research Centre, Birmingham B15 2TT, UK; Institute of Translational Medicine, University Hospitals Birmingham NHS Foundation Trust, Birmingham B15 2TT, UK; MRC Health Data Research UK (HDR), Midlands Site B15 2TT, UK

## Abstract

**Motivation:**

Existing microbiome-based disease prediction relies on the ability of machine learning methods to differentiate disease from healthy subjects based on the observed taxa abundance across samples. Despite numerous microbes have been implicated as potential biomarkers, challenges remain due to not only the statistical nature of microbiome data but also the lack of understanding of microbial interactions which can be indicative of the disease.

**Results:**

We propose CACONET (classification of Compositional-Aware COrrelation NETworks), a computational framework that learns to classify microbial correlation networks and extracts potential signature interactions, taking as input taxa relative abundance across samples and their health status. By using Bayesian compositional-aware correlation inference, a collection of posterior correlation networks can be drawn and used for graph-level classification, thus incorporating uncertainty in the estimates. CACONET then employs a deep learning approach for graph classification, achieving excellent performance metrics by exploiting the correlation structure. We test the framework on both simulated data and a large real-world dataset pertaining to microbiome samples of colorectal cancer (CRC) and healthy subjects, and identify potential network substructure characteristic of CRC microbiota. CACONET is customizable and can be adapted to further improve its utility.

**Availability and implementation:**

CACONET is available at https://github.com/yuanwxu/corr-net-classify.

**Supplementary information:**

Supplementary data are available at *Bioinformatics* online.

## 1 Introduction

Increasing evidence suggests that microbiota plays a critical role in the prognosis, diagnosis and treatment of human diseases. In particular, alterations in the gut microbiome community have been implicated in many conditions, including autoimmune diseases (De Luca and Shoenfeld, 2019; Scher and Abramson, 2011), obesity related to metabolic disorders (Vallianou *et al.*, 2019), neurodegenerative diseases (Fang *et al.*, 2020; Westfall *et al.*, 2017) and cancers, for example colorectal cancer (CRC) (Ahn *et al.*, 2013; Song *et al.*, 2020; Zackular *et al.*, 2014). The improvement and accessibility of high-throughput metagenomic sequencing technologies, the growing volume of various biological databases, and the development of software tools to facilitate their analysis have enabled researchers to identify and annotate more species, metabolites and gene products present in the gut ecosystem. Microbiome-based predictive analyses seek to understand the associations between microbiome compositions and some phenotypic outcome, such as the health status of subjects from which the samples were collected, or other covariates of interest. Microbiome profiling typically results in an operational taxonomic unit (OTU) table representing taxa abundances across samples. Such tables may be obtained by OTU clustering (Hao *et al.*, 2011; Nguyen *et al.*, 2016) of 16S rRNA sequences resulting from next-generation amplicon sequencing. Compared to classical differential abundance analysis of microbiome data, machine learning methods are often used to provide superior discriminative power, although for some class of methods the effect of microbial abundance level on the outcome variable and an importance ranking based on some measure of importance can be determined. For example, there have been numerous research efforts attempting to identify predictive omics-biomarkers which distinguish between CRC and healthy subjects, using logistic regression (Clos-Garcia *et al.*, 2020), random forest (Sze *et al.*, 2017; Yachida *et al.*, 2019) and Bayesian methods (Bisht *et al.*, 2021; Koslovsky *et al.*, 2020). In such predictive studies, although decent classification accuracy and identification of important microbes or other biomarkers that are either enriched or depleted are typically feasible, how changes in the abundance of these microbes affect the behavior of other microbes within the wider microbial community remains largely unknown. Here, ‘community’ is interpreted in the sense of ecological community, where a group of potentially interacting species live in a shared environment and form various ecological relationships such as mutualism and competition, among others (Faust and Raes, 2012). It has been demonstrated that diverse microbial interactions exist in various body sites of the human microbiome (Faust *et al.*, 2012). Therefore, despite numerous microbes have been shown to display differential abundance in cases compared to controls, or vice versa, their use as potential therapeutic targets for disease management is still hindered by the lack of understanding of their interactions with other microbes within the community.

In order to capture microbial associations, we built correlation networks from abundance data. Such networks can be treated as a weighted graph in which nodes are microbes and edge weights between pairs of microbes correspond to the correlation coefficient of their abundances. Microbial correlation networks are also referred to as co-abundance networks (Chen *et al.*, 2020). We generated a collection of correlation networks that are representative of the underlying phenotypic outcome using a compositional-aware hierarchical Bayesian method, and proposed CACONET (classification of Compositional-Aware COrrelation NETworks), a novel classification framework based on correlation structures inferred from samples of different population types. In addition, we propose an intuitive explanation method which can be used to elucidate predictions by identifying important links and nodes that contribute to the predicted class of the correlation network in question, characterizing not just individual microbes as potential biomarkers for disease, but, perhaps more importantly, delineating the context in which their interaction with other microbes manifest. Our method performs classification on entire correlation networks and attempts to learn characteristic graph signatures that differentiate phenotypic outcomes. We demonstrate its capability first through simulated datasets of varying degree of complexity, and second, by applying to an integrated fecal microbiome data comprising thousands of CRC cases and healthy controls.

## 2 Materials and methods

### 2.1 Classification framework

**Fig. 1.**
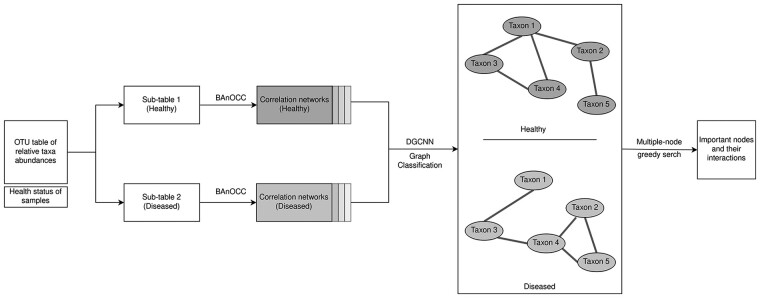
Overview of CACONET, a deep learning-based graph classification framework for microbial co-abundance networks. An OTU table of taxa relative abundances and associated phenotype or sample covariate of interest (e.g. health status), are given as input. The OTU table is then subdivided according to the phenotype and BAnOCC run to infer correlations between taxa for each sub-table, resulting in distributions of correlation networks for each phenotype. These networks are combined to form the training and testing data for the DGCNN algorithm, which learns graph signatures to differentiate between different phenotypes. To explain the prediction of the algorithm, a greedy search strategy is employed to find important nodes and interactions

The input to our proposed classification framework consists of an OTU table of relative abundances of taxa across samples and the phenotypic outcome of interest associated with the samples (e.g. healthy or diseased subjects from which the samples were taken). Such settings can be straightforwardly extended to encompass multiple outcomes or population types, such as those derived from samples at distinct cancer stages, for which interests may be to uncover distinct microbial interactions from each stage. However, in the current work, we restrict our approach to binary outcomes (healthy and disease) throughout. Due to sample variations and technological limitations in sensitivity, relative abundances instead of raw read counts were used. This is obtained by dividing by the library size of the corresponding sample (total species abundances sequenced in the sample). To avoid confusion, we refer to rows of an OTU table as samples and columns as features, taxa or microbes. The OTU table was split into sub-tables corresponding to healthy and diseased subjects, and Bayesian correlation inference was applied to each sub-table to infer putative correlations among the microbes. Rather than producing a single correlation network as a summary of the posterior distribution, we collect many networks as draws from the posterior distribution once equilibrium regime is reached, thereby incorporating posterior uncertainty into subsequent analysis. To ensure data balance, we collect an equal number of networks from the posterior distributions corresponding to healthy and diseased populations. These correlation networks were subsequently combined to form the input to a graph classification algorithm, which attempts to distinguish ‘diseased’ microbial networks from ‘healthy’ microbial networks by learning the rich structural information encoded in these graphs. We used BAnOCC (Bayesian Analysis of Compositional Covariance) (Schwager *et al.*, 2017) to infer correlations between unobserved log absolute feature abundance from compositional data, and DGCNN (Deep Graph Convolutional Neural Network) (Zhang *et al.*, 2018) to classify microbial correlation networks into distinct population types. The pipeline of our proposed approach is shown in Figure  1.

### 2.2 Microbial correlation network inference

After normalizing feature abundance in an OTU table by an unconstrained sum for each sample, the construction of correlation networks must account for the compositional nature of the data, since the additional sum-constraint (proportions must sum to one) implies that if the abundance of one microbe increases, the abundance of some other microbe must decrease to compensate the effect. Thus, traditional correlation measures (e.g. Pearson or Spearman correlation coefficient) may not be reliable and compositional-aware methods are needed. To achieve this, we used BAnOCC to infer the log absolute abundance correlation matrix.

Suppose we are given an OTU table **X** with absolute abundances, then the relative abundance of taxon *j* in sample *i* in the normalized OTU table **C** is given by $C_{ij}=X_{ij}/\sum_{j=1}^{p}X_{ij}$, where *p* is the number of taxa. We wish to infer the absolute abundance covariance matrix $\Sigma_{x}$ from **C**. BAnOCC estimates a sparse log-basis precision matrix instead, that is, $O:=\Sigma_{\;\text{log}\;x}^{-1}$. By inverting this matrix, we obtain the log-basis covariance matrix and from it the log-basis correlation matrix $R_{\;\text{log}\;x}$. Let $R_{x}$ denote the absolute abundance correlation matrix, then the existence and direction of feature associations in $R_{\;\text{log}\;x}$ match those in $R_{x}$ (Schwager *et al.*, 2017). The latent count data are modeled using a log-normal distribution, with a normal prior for its mean and a graphical LASSO prior (Wang, 2012) for its precision matrix. A Gamma hyperprior is placed on the LASSO shrinkage parameter *λ*. Let $X_{i}$ be the *i*’th row of **X**, $m:=E[\text{log}\;X_{i}]$ the log-basis mean and $S:=\Sigma_{\;\text{log}\;x}$ its covariance matrix, then the hierarchical Bayesian model BAnOCC is specified by

$$\begin{array}{c} X_{i}\;\overset{\mathrm{iid}}{∼}\;\;\text{Log-normal}(m,S)\\ m∼N(n,L)\\ S=O^{-1},\;O_{jj}∼\;\text{Exp}(\frac{\lambda }{2}),\;O_{jk}∼\text{Laplace}(\lambda )\\ \lambda ∼\text{Gamma}(a,b) \end{array}$$

where *a*, *b*, **n**, **L** are hyperparameters. Inference is done via Markov Chain Monte Carlo, in particular, the No-U-Turn Sampler for Hamiltonian Monte Carlo (Hoffman and Gelman, 2014). BAnOCC parameter specifications are provided in Supplementary Materials. The R package banocc (Schwager and Huttenhower, 2020) (version 1.14.0) was used.

It has been shown that BAnOCC performed relatively well in limiting the number of spurious correlations compared to other compositionally aware correlation methods (Schwager *et al.*, 2017). In addition, one advantage of incorporating BAnOCC into CACONET is that we are able to take the uncertainty of correlation estimates into consideration and specify prior assumptions about sparsity through the sparsity parameter. Instead of summarizing a single correlation network by using, for example, posterior median correlations, we treat the posterior as a generator and draw many networks from it. These networks can be identified with the original population type (Healthy or Disease) associated with the OTU table, and the posterior uncertainty reflects the uncertainty of microbial co-abundance pattern within that population type.

### 2.3 Microbial correlation network classification

In order to distinguish microbial correlation networks representative of the healthy population from that of the diseased population, we used the DGCNN algorithm (Zhang *et al.*, 2018). DGCNN was designed for graph-level classification where a collection of labeled graphs as well as a matrix providing node-level information are given as input (such a matrix is often called ‘node feature matrix’ in machine learning literature, but since we have used ‘feature’ to mean a taxon or microbe, we refer to this matrix as ‘node information matrix’ instead), and the task is to learn to assign the graphs to their correct categories. DGCNN uses graph convolution layers designed to extract rich structural information encoded in a graph and is shown to perform competitively across various benchmark datasets and other graph neural network methods (Zhang *et al.*, 2018). It operates with the following layer propagation rule:

$$Z^{\left(l+1\right)}=\sigma ({\tilde{D}}^{-1}\tilde{A}Z^{\left(l\right)}W^{\left(l\right)})$$

where $\tilde{A}=A+I$, the adjacency matrix *A* with added self-loops, ${\tilde{D}}_{ii}=\sum_{j}{\tilde{A}}_{ij}$ the diagonal degree matrix, $Z^{\left(0\right)}=X$ the node information matrix, $W^{\left(l\right)}$ the layer-specific trainable weight matrix and *σ* is some nonlinear activation function. The matrices of activations ${\left\{Z^{\left(l\right)}\right\}}_{l=1}^{h}$ encode multi-scale local structure for the nodes, with the final layer activation $Z^{\left(h\right)}$ representing the most refined partition of the nodes in the graph. These matrices ${\left\{Z^{\left(l\right)}\right\}}_{l=1}^{h}$ are concatenated horizontally for which a SortPooling layer is applied in order to sort the nodes in a consistent manner. Specifically, it rearranges the nodes in descending order based on the last channel of $Z^{\left(h\right)}$. The SortPooling layer is then followed by several 1-D convolution and MaxPooling layers and finally a fully-connected and softmax layer. Here, we used the adjacency matrix for weighted graphs where *A_ij_* is the posterior correlation between taxa *i* and *j* inferred by BAnOCC. We took *X* to be the identity matrix because (i) there is limited knowledge about the microbes other than their abundances, and (ii) it allows us to test whether the predictive power of CACONET can be attributed to correlation structure alone. We list hyperparameter choices used in our study in Supplementary Materials. The Python library StellarGraph (CSIRO’s Data61, 2018) (version 1.2.1) was used for implementation.

### 2.4 Elucidating biological insights from network classification

A good classification accuracy is a strong indication of the presence of distinctive graph signatures. However, the distinctive features of the correlation networks, especially those indicative of diseased samples, are arguably more interesting and valuable to biomedical researchers, since they provide insights into potential interactions of specific microbes. Such interactions may be characteristic of the diseased microbiota and thus may serve as targets for intervention and treatment of the disease, potentially, for example, by disrupting those signature interactions. It is therefore important to understand how CACONET has learned to make a prediction on the class of a network. Unfortunately, most explainable artificial intelligence methods developed for convolutional neural networks, such as SmoothGrad (Smilkov *et al.*, 2017) and GradCAM (Selvaraju *et al.*, 2017), are designed for computer vision applications aiming to generate images with well-defined human interpretable semantics. However, these approaches would not be feasible for graphs because the differences between two densely connected graphs are difficult to be interpreted by humans.

In order to extract important signatures in the graph, we used an intuitive approach of ‘knocking-out’ nodes in the graph and examine how the prediction probability changes. Specifically, for a given graph, we set all edge weights incident to one node to zero, that is, we set this node to be uncorrelated with all other nodes, we then compute the log-odds ratio (LOR) of the new graph relative to the original one. The LOR is defined as

$$\text{log}\;(\frac{p_{w_{v}:=0}}{1-p_{w_{v}:=0}})-\text{log}\;(\frac{p}{1-p}),$$

where *p* denotes the prediction probability of the ‘diseased’ class and $p_{w_{v}:=0}$ the new probability when all edge weights linking node *v* are set to 0. This procedure is successively applied to each node in the graph and the node which results in the largest change in log odds is likely to be important. Similarly, we could consider knocking-out all possible combinations of two or more nodes and select the node set with the largest LOR. However, such an approach would be computationally infeasible due to combinatorics of the problem. We therefore devised a greedy algorithm (Algorithm 1) as follows: assume the most important node has been found by the above procedure, the next node is to be searched from the list of the remaining nodes and the one which results in the largest absolute LOR is added, forming the most important node pair. Continuing in this fashion, the top *n* most important nodes (*n*-node) can be found. In Algorithm 1, with an abuse of notation, we have used $p_{v:=0}$ to denote the probability of the graph when all edges linking to node *v* are set to zero weights.

Algorithm 1 *n*-node importance

**Require:**  n≥1
 

$$v_{1}\leftarrow {\text{argmax}}_{v\in V}\left|\;\text{log}\;(\frac{p_{v:=0}}{1-p_{v:=0}})-\text{log}\;(\frac{p}{1-p})\right|$$

 **if** *n *=* *1 **then       return** *v*_1_**  end if  for**  i in 2:n  **do**

**    **

$v_{i}\leftarrow {\text{argmax}}_{v\in V\setminus \{v_{1}\ldots v_{i-1}\}}\left|\;\text{log}\;(\frac{p_{\{v_{1}\ldots v_{i-1}\}\cup v:=0}}{1-p_{\{v_{1}\ldots v_{i-1}\}\cup v:=0}})-\text{log}\;(\frac{p}{1-p})\right|$

**  **Add *v_i_* to output.

 **end for**

### 2.5 Simulation study

The recently proposed SparseDOSSA (Sparse Data Observations for the Simulation of Synthetic Abundances) (Ma *et al.*, 2021) for microbiome data fitting and simulation allows us to simulate realistic synthetic microbial profiles with the added flexibility of controlling microbe–microbe and microbe–environment associations. It adopts a hierarchical statistical framework that attempts to address challenging characteristics of microbiome data such as zero-inflation, high-dimensionality and feature dependency. SparseDOSSA models the marginal distribution of absolute (pre-normalized) feature abundance as a zero-inflated log-normal distribution, thus taking into account biological as well as technical absences. A multivariate Gaussian copula specifies the correlation structure among the features. The model likelihood is optimized via a penalized expectation-maximization method. Once fitted, new microbial features can be simulated using the fitted parameters. Here, we simulated artificial taxa that resembled the pre-trained stool template dataset using the R package SparseDOSSA2 (version 0.99.2).

In order to simulate known covariance structures between taxa, we followed Ma *et al.* (2021) by letting pairs of taxa associate with a standard normal covariate (association spike-in). This was done using (generalized) linear models for both non-zero abundance,

$$\mu_{j}^{\prime }=\mu_{j}+\beta Z,\;\mu_{k}^{\prime }=\mu_{k}+t\beta Z$$

and prevalence,

$$\text{log}\;\frac{1-\pi_{j}^{\prime }}{\pi_{j}^{\prime }}=\text{log}\;\frac{1-\pi_{j}}{\pi_{j}}+\beta Z,\;\;\text{log}\;\frac{1-\pi_{k}^{\prime }}{\pi_{k}^{\prime }}=\text{log}\;\frac{1-\pi_{k}}{\pi_{k}}+t\beta Z,$$

where $\mu_{j},\mu_{k}$ are the mean non-zero log absolute abundances for features *j* and *k*, $\pi_{j},\pi_{k}$ are the absence probabilities for features *j* and *k*. $\mu_{j}^{\prime },\mu_{k}^{\prime },\pi_{j}^{\prime },\pi_{k}^{\prime }$ are the new mean log absolute abundances and absence probabilities for the corresponding features. *β* can be interpreted as effect size that determines the strength of correlation, with t∈{−1,1} specifying the desired direction: positive if *t *=* *1 and negative if *t* = – 1. *Z* is standard normal random variable. Feature-feature associations with varying effect sizes and directions can then be simulated through this procedure.

**Fig. 2.**
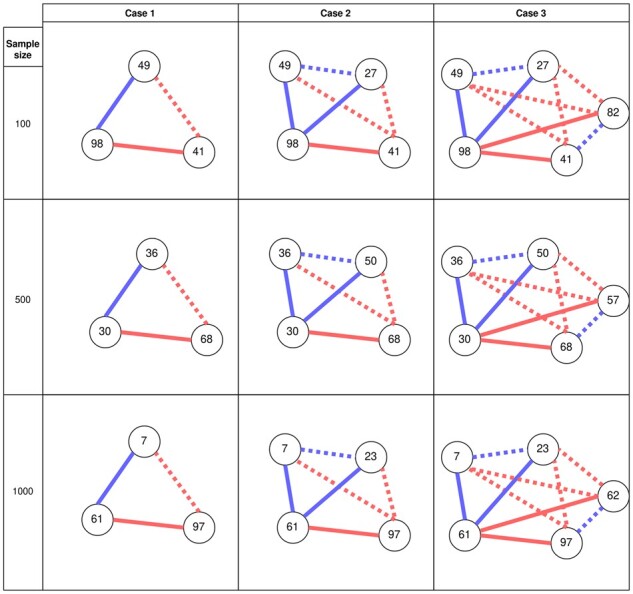
Three scenarios (Cases 1–3) of correlation structure with increasing complexity across different sample sizes, used as hypothesized signatures in the ‘disease’ population. For Case 1, two pairs of features were enforced to have a positive and negative correlation, with a common feature (Feature96 for sample size 100, Feature30 for sample size 500 and Feature61 for sample size 1000) present in both pairs. For Cases 2 and 3, three and four pairs of features respectively were enforced to have alternating positive and negative correlations, again with a common feature present in all feature pairs. Such a simulation setup would cause feature pairs not spiked-in with a correlation to be correlated, namely induced correlations. Solid lines indicate correlations that were enforced and dotted lines indicate induced correlations. Positive correlations are shown in blue and negative ones in red

We simulated different sample sizes (100, 500, 1000), each with 100 features. For each sample size, three case groups (hypothetical diseased groups) with increasing complexity, were considered. For each sample size, we first performed a baseline simulation with no association spike-in and selected the top few most abundant features present in at least 50% of samples. For example, assuming Feature1 to Feature5 were the five most abundant features for sample size 100, then, for Case 1, we enforced positive and negative correlations for feature pairs (Feature1, Feature2) and (Feature1, Feature3), respectively, resulting in an induced negative correlation between Feature2 and Feature3; for Case 2, we added another feature pair (Feature1, Feature4), and enforced positive correlation between them, resulting in two more induced correlations; for Case 3, we enforced negative correlation to yet another feature pair (Feature1, Feature5). We used the default effect size *β *= 1 in all simulated cases. The most abundant features used for association spike-in were the same within each sample size but may differ across sample sizes. The simulated correlation structure for all sample sizes and case groups are illustrated in Figure  2. For the control groups, we simply repeated the simulation procedure for each sample size but without association spike-in.

For each sample size and case number, features with relative abundance >0.0001 in at least 30% of samples in the combined control and case group simulated OTU table were kept. We were thus able to simulate the challenging scenario of the presence of complete subgraphs of varying complexity and heterogeneous microbe–microbe interactions in microbial ecology.

The simulated data were converted to relative abundance to make it compatible with compositional input and was fitted with BAnOCC until convergence. The posterior correlation networks inferred from each case group were combined with those from the control group and graph-level classification proceeded as in Figure  1. With this simulation setup, we would expect that the nodes selected by the greedy search are likely to be among the ones present in the respective cases in Figure  2, and that the common feature chosen for spike-in (Feature98, Feature30, Feature61 for sample sizes 100, 500, 1000, respectively) is likely to be the most important node found by the single-node knock-out approach.

### 2.6 Real-world example

In order to assess the ability to classify microbial correlation networks with real-world data and to explore important microbes within such networks, we applied CACONET to the Microbiome Quality Control (MBQC) project (Sinha *et al.*, 2015) baseline data pertaining to health status of either healthy or CRC case. MBQC is a collaborative project aimed to assess the reproducibility of microbiome studies across various sources of variations from sample collection, storage, DNA extraction, sequencing and bioinformatics protocols. The project involved 16 sample handling and 9 bioinformatics laboratories. In its baseline phase only 16s rRNA amplicon profiling and analyses of the human fecal microbiome were performed. The resulting baseline data included raw sequences, integrated OTU tables and other data products.

We took the integrated OTU table of the baseline data (available from https://www.hmpdacc.org/MBQC/), selected ‘Fresh’ and ‘Freeze-dried’ specimen types, and filtered samples with health status of either ‘CRC case’ or ‘Healthy’. Taxa were agglomerated to genus level and those present in at least 40% of samples with relative abundance >0.0001 were kept. Taxa with unclassified genus were identified with the next available higher rank; for example, if a taxon has an unclassified genus but a classified family, the family rank would be used to identify that taxon. Samples with zero sum of taxa abundance were removed, resulting in 5232 samples with 2713 CRC and 2519 healthy samples. The resulting OTU table with relative abundances was split into sub-tables corresponding to healthy and CRC. A total of 800 correlation networks were drawn separately from the respective posterior distribution, forming the input to the graph classification algorithm with perfectly balanced control and case groups (800 Healthy and 800 CRC). We performed 10 repeated DGCNN runs with 100 epochs per run, each time trained on a different random split of the data (80-20 training-validation split). *n*-node importance calculation with *n* up to 21 was performed based on Algorithm 1 on a random subsample of the graphs and aggregated over all DGCNN runs.

## 3 Results

### 3.1 Simulated data

We tested the ability of CACONET to distinguish networks with a particular association spike-in from those that do not have such signatures across various sample sizes and correlation complexity. Furthermore, we assessed how well the pre-spiked features can be recovered using the feature knock-out and greedy search strategy.

We performed 10 DGCNN runs, each with 80 epochs, and achieved excellent validation performance across all sample sizes and case numbers (Table  1). In general, we observed an improvement in all metrics as sample size increases. Within each sample size, the mean accuracy increased as the correlation structure became more complex, which can be explained by the observation that an increase of the features used for association spike-in directly correlates with the algorithm’s ability to distinguish more complex networks from ones without association spike-in. When some spiked-in associations were not recognized by the algorithm, other spiked-in associations may be sufficient for making a correct prediction. Interestingly, in the most complex case (Case 3), the performance metrics were consistently good across all sample sizes, achieving over 99% accuracy. The training and validation trajectories of all metrics were listed in Supplementary Figures S1–S3.

**Table 1.** Performance metrics evaluated on the validation set of the simulated correlation networks, for all sample sizes and case numbers corresponding to Figure  2

		Accuracy		Precision		Recall		AUC	
		Mean	Std	Mean	Std	Mean	Std	Mean	Std
Sample size	Case								
**100**	**1**	0.9645	0.0148	0.9726	0.0164	0.9560	0.0184	0.9944	0.0038
	**2**	0.9765	0.0236	0.9846	0.0166	0.9680	0.0358	0.9956	0.0071
	**3**	0.9940	0.0032	0.9931	0.0066	0.9950	0.0071	0.9994	0.0017
**500**	**1**	0.9960	0.0046	0.9960	0.0052	0.9960	0.0052	0.9994	0.0016
	**2**	0.9975	0.0035	0.9980	0.0042	0.9970	0.0067	1.0000	0.0001
	**3**	0.9950	0.0033	0.9980	0.0042	0.9920	0.0042	0.9989	0.0022
**1000**	**1**	0.9875	0.0155	0.9899	0.0142	0.9850	0.0190	0.9994	0.0016
	**2**	0.9980	0.0042	0.9990	0.0032	0.9970	0.0067	0.9999	0.0002
	**3**	0.9995	0.0016	0.9990	0.0031	1.0000	0.0000	1.0000	0.0000

*Note*: Each metric was averaged over 10 DGCNN runs, with mean and standard deviation shown.

**Fig. 3.**
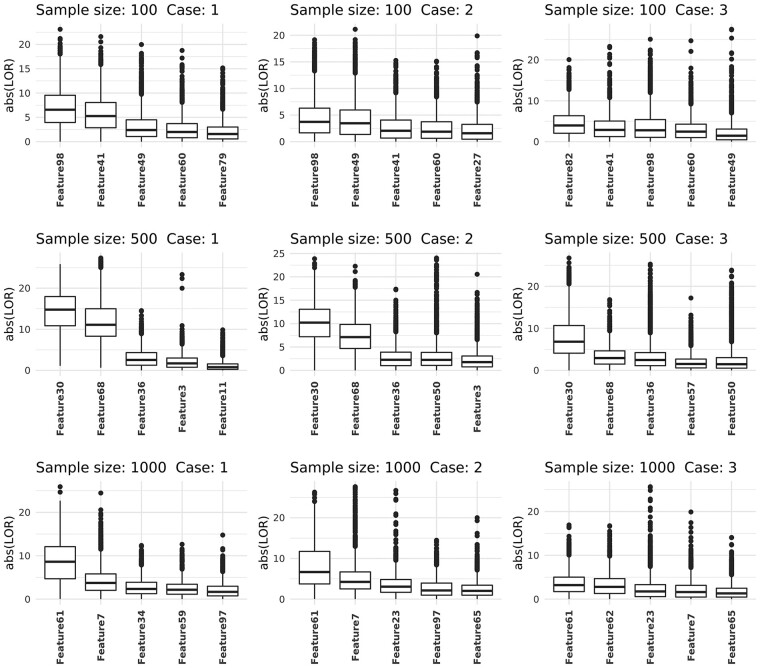
Box-and-whisker plot of single-node importance measured as change in log-odds of prediction when a node is ‘knocked-out’ from the correlation network (all correlations with the node in question are set to zero), shown in decreasing order of median absolute LOR for five nodes for each sample size and case number corresponding to Figure  2. LOR was calculated from a subsample of the networks and pooled from all DGCNN runs

Figure  3 shows the distribution of the absolute LOR for those nodes resulting in the largest median LOR in single-node knock-out. Except for Case 3 with sample size 100, the common features used to enforce associations (Feature98, Feature30 and Feature61) had the largest median absolute LOR in all other configurations, although there was substantial overlap in distribution across all features in Case 3 with sample sizes 100 and 1000. This result confirms that the most important node can be found by the single-node knock-out approach. However, when the number of feature-feature associations is large, as is typically the case in real microbial ecology, knocking-out a single node may not have an effect large enough to alter the nature of the network, and the remaining associations present in the network may still impart a strong signal.

**Fig. 4.**
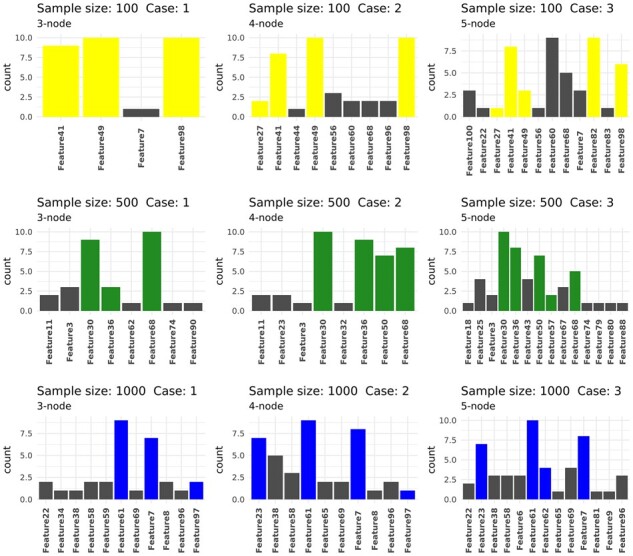
Frequency of occurrence of the k(k=3,4,5) most important nodes across 10 DGCNN runs, for each sample size and case number. For each run, a greedy search was used to find *k* most important nodes. These nodes were aggregated over all runs and their frequencies of occurrence were shown. Pre-spiked nodes (Fig.  2) were highlighted for sample size 100 (yellow), 500 (green) and 1000 (blue). In general, the more frequent nodes agree with those used for association spike-in, with some exceptions such as Feature60 and Feature68 in Case 3 with sample size 100. We also note that Feature97 was not seen in Case 3 with sample size 1000, despite being one of the features used for spike-in. A close examination of the fitted posterior median correlation network for this case (Supplementary Fig. S4) revealed that Feature97 was not shown to be correlated with any other features, suggesting that BAnOCC did not have sufficient power in this particular case

We now turn to multiple-node importance using the greedy search heuristic. We show the frequency of occurrence of *k* most important nodes over all DGCNN runs, where *k* corresponds to the number of nodes chosen for association spike-in in each case (*k *=* *3, 4, 5 for Cases 1 to 3, respectively, see Fig.  2). In general, the pre-spiked nodes appeared more frequently than nodes not used for spike-in (Fig.  4), and thus our search heuristic can be used to find a collection of nodes jointly influencing the graph prediction. However, the stability of the found nodes may be improved by multiple iterations of the algorithm.

Supplementary Figure S4 shows the fitted median correlation networks for each scenario in Figure  2. Edges with median correlation strength >0.2 were shown. For sample size 500, Cases 2 and 3 perfectly reconstruct the core correlation structure shown in Figure  2, whereas (Feature98, Feature79) in Case 1 of sample size 100, (Feature98, Feature83) in Case 3 of sample size 100 and (Feature61, Feature38) in Case 3 of sample size 1000, are examples of false positive correlation pairs that were not present in the original configurations.

### 3.2 Real-world data from the Microbiome Quality Control Project

CACONET achieved near-perfect performance on the MBQC data (average accuracy 99.97%, precision 100%, recall 99.94%). Supplementary Figure S5 shows various metrics over the number of epochs.

We found that only two microbes resulted in a slightly above zero median absolute LOR: 0.029 for *Parabacteroides* and 0.0077 for *Sutterella*; the rest of the microbes have essentially zero effect on network prediction (median absolute LOR $1.46\times 10^{-7}$). This result suggests that the CRC microbial correlation network is relatively robust to any single-node perturbation. The implication is that interventions targeting at any single microbe are unlikely to result in significant improvement in the health outcome.

**Fig. 5.**
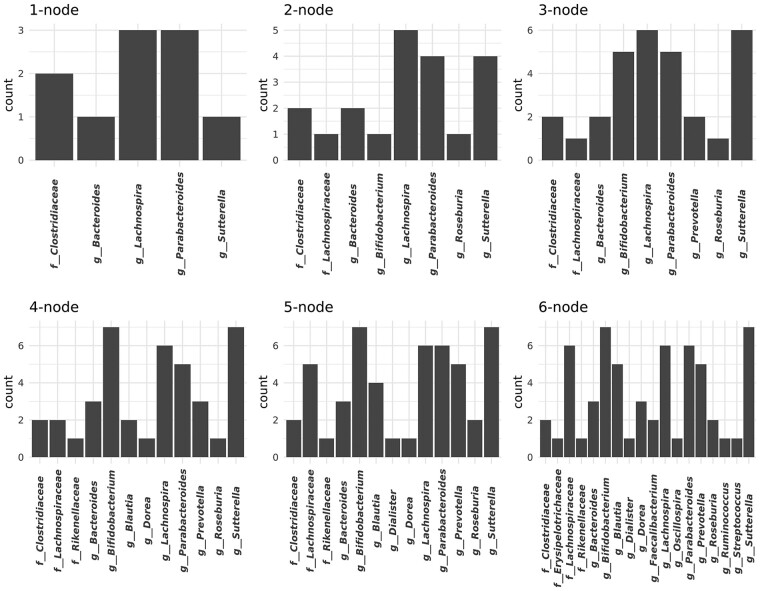
Frequency of occurrence of the *k* most important nodes (*k*-node) across all DGCNN runs. For each k, (k=1…6), the top *k* nodes resulting in the largest median absolute LOR were selected and pooled across all runs. The number of times each microbe appeared in the pool was shown in the vertical axis. As each new run may produce a *k*-node that only partially overlap with those *k*-node obtained from existing runs, the number of microbes shown in each panel of the plot encompasses all occurrences and hence is greater than *k*. While in the 1-node case the difference in count between the most frequent and the least frequent microbe is only 2, the difference increases to 6 when more than four nodes are considered

To investigate the effect of simultaneously knocking out multiple nodes on the prediction of the graph, we show in Figure  5 the frequency of occurrence of *k* most important nodes in predicting CRC correlation networks, for *k* from 1 to 6. As an example, for *k *=* *1 (1-node), the microbe that resulted in the largest median absolute LOR per DGCNN run was selected, and the number of times it appeared in all runs was shown in the vertical axis of Figure  5. Because of the nature of the greedy search, microbes appearing in the *k* most important nodes must also appear in the *k *+* *1 most important nodes, that is, *k*-node is a subset of (k+1)-node. It can be seen that the frequency of occurrence for 1-node are more evenly distributed among the microbes, confirming our observation of the neglecting effect of any single microbes. However, as *k* increases, certain microbes appear considerably more frequently than others, suggesting that they may play an important role in the characterization of CRC microbial networks.

In order to examine to what extent the LOR changes with the number of knocked-out nodes, *k*, we plotted the median absolute LOR as a function of *k* for all DGCNN runs (Supplementary Fig. S6). As expected, the magnitude of LOR increases as *k* increases, however, the rate of increase slows down as *k* becomes larger, suggesting the existence of a critical number of nodes potentially forming the core structure of the CRC microbial correlation network. When this core structure is destroyed, the network likely losses most of its CRC characteristics.

**Fig. 6.**
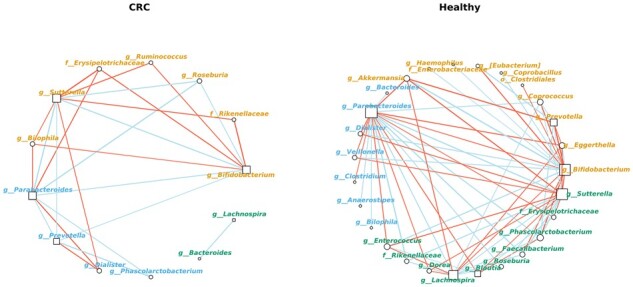
Representative correlation networks inferred from CRC (left) and Healthy (right) samples extracted from the MBQC baseline integrated OTU table. The microbes selected from 6-node importance (Fig.  5) were shown in square, and those with whom the correlation strength was >0.4 were shown in circle. A community detection algorithm attempting to maximize a modularity measure over all possible partitions was run using the cluster_optimal routine of the R package igraph (Csardi and Nepusz, 2006), and the resulting clusters were identified with the color of the node label. Positive correlations are shown in blue and negative correlations in red, the thickness of the line is proportional to the correlation strength. Node size is proportional to the square root of its degree

To assess how the CRC correlation network might differ from that derived from the healthy subjects, we selected all microbes appearing in at least half of the runs in the 6-node panel in Figure  5, corresponding to those with counts at least five, namely bacterial genera *Bifidobacterium*, *Blautia*, *Lachnospira*, *Parabacteroides*, *Prevotella*, *Sutterella*, and bacterial family *Lachnospiraceae*. We then extracted the part of the correlation network involving strong correlations with these microbes separately for CRC and Healthy (Fig.  6). In both cases, the network represents a summary of the posterior distribution in which edges with absolute median correlation >0.4 are shown. A community detection algorithm was used to partition the network into cohesive groups or clusters, called ‘network communities’ in graph terminology. We shall use ‘cluster’ to refer to such an entity so as to distinguish it from an ecological community.

In both networks, we observed three clusters indicated by the node label color. However, there was considerable difference in the number of microbial associations between clusters, namely the number of inter-cluster links. There were more inter-cluster links in the Healthy network than the CRC network. In addition, we found a cluster in the CRC network formed by the genera *Bacteroides* and *Lachnospira* with no strong associations with other microbes, in contrast to the Healthy network in which all clusters have inter-cluster links. There were 13 nodes and 25 edges present in the CRC network, compared to 27 nodes and 68 edges in the Healthy network. This finding is in agreement with some clinical studies of the interplay between microbiome and cancers, which suggests a greater species diversity in healthy individuals compared to the microbial ecology of people with certain diseases (Ley, 2010; Ma *et al.*, 2019), and that high species diversity likely promotes healthy competition among microbes and maintains stability of the gut community (Johnson and Burnet, 2016). It is also consistent with a meta-analysis of gut microbiome studies across diseases including CRC and several others, which suggests a decreased prevalence of bacteria non-specifically associated with disease compared with bacteria non-specifically associated with health (Duvallet *et al.*, 2017).

In addition, we observed an increased number of connections of *Lachnospira*, *Parabacteroides*, *Bifidobacterium* and *Sutterella* with other microbes in the Healthy network, compared with the CRC network. Of particular interest is *Lachnospira*, which had many strong associations in the Healthy network but was only associated with *Bacteroides* in the CRC network. Interestingly, this association with *Bacteroides* was not present in the Healthy network. This result suggests that *Lachnospira* may play an important role in regulating the gut microbiota. A key component of CRC microbial community could be characterized by the break-down of the diverse *Lachnospira* interactions and the emergence of new association with *Bacteroides*. It is also worth noting that *Blautia* was associated with several microbes in the Healthy network, including *Enterococcus*, *Parabacteroides*, *Phascolarctobacterium* and *Sutterella* but was abscent in the CRC network. The potential probiotic functions of *Blautia* have been recently reviewed in Liu *et al.*, 2021, whereas *Parabacteroides* has been associated with both probiotic and pathogenic roles in human health (Ezeji *et al.*, 2021).

It has previously been described that *Lachnospira* and *Bacteroides* exist in a symbiotic relationship and have key roles in ameliorating inflammation and supporting coloncyte health (Jia *et al.*, 2021). However, it is unknown why this relationship may only be present in the CRC network. The *Lachnospira* genus is primarily responsible for the fermentation of pectin (Cotta and Forster, 2006), which results in short chain fatty acid (SCFA) production (Bang *et al.*, 2018). SCFAs play a vital role in the promotion of a healthy microbiome and prevention of CRC (Hinnebusch *et al.*, 2002), which may explain the significant number of *Lachnospira* connections in the healthy model. *Blautia* has similar roles in SCFA production, though predominantly produces propionate and propanol (Vacca *et al.*, 2020). Hence, depletion of *Lachnospira* and/or *Blautia* in CRC may result in a decreased SCFA production, which may be responsible for reduced interactions with other species in CRC patients (Bisht *et al.*, 2021).

## 4 Discussion

Existing studies employing machine learning to identify predictive microbiome biomarkers traditionally rely on sample-level feature abundance data. As such, the effectiveness of these methods is degraded by a limited sample size, which can be much smaller than the number of features. The relative nature of feature abundance data creates another layer of complexity for statistical analysis. Various transformation strategies have been proposed to mitigate the issues caused by compositionality, such as the centered log-ratio transformation, the isometric log-ratio transformation (Egozcue *et al.*, 2003) and more complex approaches based on balances (Quinn *et al.*, 2020; Rivera-Pinto *et al.*, 2018). Our proposed framework, however, classifies not on samples, but on the correlation networks derived from samples of different population types. Thus, it is possible to generate any number of networks for classification provided the posterior distributions have reached equilibrium. As such, the accuracy of correlation inference from relative abundance forms the main limiting factor. Here, we used BAnOCC since it has been shown to perform well in the presence of spurious correlations (Schwager *et al.*, 2017), and, to our knowledge, is the only Bayesian method for compositional correlation inference currently available. We note that the tested scenarios in Schwager *et al.* (2017) included only distinct feature pairs, whereas the simulated cases considered in our study included one feature interacting with multiple other features. As pointed out in the original paper, BAnOCC could be further improved, for example by incorporating zero-inflation into the model, the absence of which could potentially explain the mismatch between the reconstructed and the ground-truth correlation networks in our simulated data. We anticipate that further BAnOCC refinements, as well as emerging Bayesian compositional correlation inference methods, will reduce the false positive and false negative rates and therefore enhance CACONET’s performance.

One related method in predictive analysis of omics data using graph convolutional neural networks is MOGONET (Wang *et al.*, 2021). In an earlier comparative study, it has been shown that kernel-based algorithms can outperform graph-based data integration algorithms in classification of binary traits (Yan *et al.*, 2017). Although multi-omics integration is not the focus of this study, there are several aspects worth highlighting in comparison with MOGONET. First, MOGONET used similarity networks where nodes are samples and edges are weighted by cosine similarity between nodes; this is similar to correlation networks used in our method, since centered cosine similarity is equivalent to Pearson correlation coefficient. However, nodes in our networks correspond to features rather than samples. As a result, MOGONET performs node-level classification whereas we seek to classify whole graphs. Second, because of this design choice, we explore associations between features rather than intra- and interclass relationship between samples, as is the case in Wang *et al.* (2021). This is substantiated by the observation that microbial interactions are ubiquitous in microbiome ecology, and signature interactions likely exist that distinguish between different population types. Third, the search strategy we proposed is somewhat similar to the ablation method used in MOGONET to identify important omics biomarkers; however, our method is able to identify multiple features jointly contribute to predictions, and hence account for feature interactions that can be present in data.

The novelty of our framework lies in the ability to make predictions not based on feature vectors, but rather on inferred correlation networks which potentially encode complex interactions. Such a transition not only renders it possible to apply advanced graph deep learning methods and to leverage efficient and highly-optimized computational framework [e.g. TensorFlow (Abadi *et al.*, 2015)], but, perhaps more crucially, allows direct learning on complex interactions between features. However, CACONET is not designed for predictions on directly observed sample data. This is a consequence of transforming the prediction target from sample-level feature abundance to population-level feature–feature correlation graphs, and so our method is best suited to detecting complex, important feature interactions characteristic of certain populations (e.g. healthy and disease), rather than predicting the health status of a new patient sample. When class imbalance is an issue, adjustments need to be made to either balance the data or properly modify the loss function, as is the case in MOGONET where different weights are assigned to the losses of different classes. Our method does not require this step because we can generate equal number of samples per class from the respective posterior distributions derived from BAnOCC, as many samples as is appropriate for analysis. However, the imbalance of the original data does affect the correlation estimation for the minority class, in which case one may consider imposing a stronger, informative prior distribution for the parameters of BAnOCC.

DGCNN has been demonstrated to exhibit excellent discriminative power, in both simulated and real-world data, suggesting the existence of distinctive graph signatures which could be potential targets for intervention. In order to better understand the prediction mechanism of the algorithm, a greedy algorithm was proposed to identify an arbitrary number of the most important nodes that jointly contribute to the predicted class, and it was shown to work reasonably well across different sample sizes and correlation complexity in the simulated data. This search strategy resembles the forward stepwise approach and therefore can be computationally expensive when the number of nodes is large.

By using MBQC baseline data to test our method, we account for inherent variations present in many individual cohort studies due to differences in handling and bioinformatics pipelines used, thus making our results more robust to changes in these protocols. Our framework can serve as a general methodology for other data types in biology and other fields, where the existence of feature interactions is suspected to be the dominating factor that distinguishes different population groups. Drawing motivations from challenges posed by microbiome data, we have applied our method to an integrated experimental dataset containing fecal micorobiome samples of CRC patients. Our results suggest a collective behavior among microbes, and the existence of a core network structure which potentially uniquely characterize CRC microbial interactions. Moreover, we found no evidence in the metadata suggesting that the patients had received any treatment before sample collection. However, we note that whilst we found no evidence in the studies included that any of the participating patients had received any chemotherapy or radiotherapy treatment prior to sample collection, our results would have been affected if that was the case since such treatments could alter the gut microbiome composition (Ervin *et al.*, 2020). Assessing our methods on other microbiome data of similar nature may be needed, and further biological insights may be revealed.

With the development of more robust and accurate correlation inference as well as explainable artificial intelligence methods easily applicable to graphs, it is anticipated that our novel classification framework can be a valuable tool for computational microbiome biomarker discovery, where not only the abundance level of individual microbes, but more importantly the unique microbial interactions characteristic of the diseased population, could play a key role in microbiome-based treatment strategy and therapeutic design.

## Supplementary Material

btaa879_Supplementary_Data

## Data Availability

The MBQC data underlying this article are available in the project website https://www.hmpdacc.org/MBQC/. The simulated data are available at https://github.com/yuanwxu/corr-net-classify.

## References

[btab879-B1] Abadi M. et al (2015). *TensorFlow: Large-Scale Machine Learning on Heterogeneous Systems*. Software available from tensorflow.org (10 December 2021, date last accessed).

[btab879-B2] Ahn J. et al (2013) Human gut microbiome and risk for colorectal cancer. J. Natl. Cancer Inst., 105, 1907–1911. 24316595 10.1093/jnci/djt300 PMC3866154

[btab879-B3] Bang S.-J. et al (2018) The influence of *in vitro* pectin fermentation on the human fecal microbiome. AMB Express, 8, 1–9. 29909506 10.1186/s13568-018-0629-9 PMC6004267

[btab879-B4] Bisht V. et al (2021) Integration of the microbiome, metabolome and transcriptomics data identified novel metabolic pathway regulation in colorectal cancer. Int. J. Mol. Sci., 22, 5763. 34071236 10.3390/ijms22115763 PMC8198673

[btab879-B5] Chen L. et al (2020) Gut microbial co-abundance networks show specificity in inflammatory bowel disease and obesity. Nat. Commun., 11, 1–12. 32782301 10.1038/s41467-020-17840-y PMC7419557

[btab879-B6] Clos-Garcia M. et al (2020) Integrative analysis of fecal metagenomics and metabolomics in colorectal cancer. Cancers, 12, 1142. 32370168 10.3390/cancers12051142 PMC7281174

[btab879-B7] Cotta M. , Forster R. (2006). The Family Lachnospiraceae, Including the Genera Butyrivibrio, Lachnospira and Roseburia. Springer US, New York, NY, pp. 1002–1021.

[btab879-B8] Csardi G. , Nepusz T. (2006) The igraph software package for complex network research. Int. J. Complex Syst., 1695, 1–9.

[btab879-B9] CSIRO’s Data61. (2018). *Stellargraph Machine Learning Library*. https://github.com/stellargraph/stellargraph (3 December 2021, date last accessed).

[btab879-B10] De Luca F. , Shoenfeld Y. (2019) The microbiome in autoimmune diseases. Clin. Exp. Immunol., 195, 74–85. 29920643 10.1111/cei.13158 PMC6300652

[btab879-B11] Duvallet C. et al (2017) Meta-analysis of gut microbiome studies identifies disease-specific and shared responses. Nat. Commun., 8, 1784. 29209090 10.1038/s41467-017-01973-8 PMC5716994

[btab879-B12] Egozcue J.J. et al (2003) Isometric logratio transformations for compositional data analysis. Math. Geol., 35, 279–300.

[btab879-B13] Ervin S.M. et al (2020) Relationship between the gut microbiome and systemic chemotherapy. Dig. Dis. Sci., 65, 874–884. 32026181 10.1007/s10620-020-06119-3 PMC7046092

[btab879-B14] Ezeji J.C. et al (2021) Parabacteroides distasonis: intriguing aerotolerant gut anaerobe with emerging antimicrobial resistance and pathogenic and probiotic roles in human health. Gut Microbes, 13, 1922241. 34196581 10.1080/19490976.2021.1922241 PMC8253142

[btab879-B15] Fang P. et al (2020) The microbiome as a modifier of neurodegenerative disease risk. Cell Host Microbe, 28, 201–222. 32791113 10.1016/j.chom.2020.06.008 PMC7430034

[btab879-B16] Faust K. , Raes J. (2012) Microbial interactions: from networks to models. Nat. Rev. Microbiol., 10, 538–550. 22796884 10.1038/nrmicro2832

[btab879-B17] Faust K. et al (2012) Microbial co-occurrence relationships in the human microbiome. PLoS Comput. Biol., 8, e1002606. 22807668 10.1371/journal.pcbi.1002606 PMC3395616

[btab879-B18] Hao X. et al (2011) Clustering 16S rRNA for OTU prediction: a method of unsupervised Bayesian clustering. Bioinformatics, 27, 611–618. 21233169 10.1093/bioinformatics/btq725 PMC3042185

[btab879-B19] Hinnebusch B.F. et al (2002) The effects of short-chain fatty acids on human colon cancer cell phenotype are associated with histone hyperacetylation. J. Nutr., 132, 1012–1017. 11983830 10.1093/jn/132.5.1012

[btab879-B20] Hoffman M.D. , Gelman A. (2014) The no-U-turn sampler: adaptively setting path lengths in hamiltonian monte carlo. J. Mach. Learn. Res., 15, 1593–1623.

[btab879-B21] Jia W. et al (2021) Gut microbiota alterations are distinct for primary colorectal cancer and hepatocellular carcinoma. Protein Cell, 12, 374–393. 32797354 10.1007/s13238-020-00748-0 PMC8106555

[btab879-B22] Johnson K.V.-A. , Burnet P.W. (2016) Microbiome: should we diversify from diversity? Gut Microbes, 7, 455–458. 27723427 10.1080/19490976.2016.1241933 PMC5103657

[btab879-B23] Koslovsky M.D. et al (2020) A Bayesian model of microbiome data for simultaneous identification of covariate associations and prediction of phenotypic outcomes. Ann. Appl. Stat., 14, 1471– 1492.

[btab879-B24] Ley R.E. (2010) Obesity and the human microbiome. Curr. Opin. Gastroenterol., 26, 5–11. 19901833 10.1097/MOG.0b013e328333d751

[btab879-B25] Liu X. et al (2021) Blautia—a new functional genus with potential probiotic properties? Gut Microbes, 13, 33525961. 10.1080/19490976.2021.1875796 PMC7872077 33525961

[btab879-B26] Ma S. et al (2021) A statistical model for describing and simulating microbial community profiles. PLoS Comput. Biol., 17, e1008913. 34516542 10.1371/journal.pcbi.1008913 PMC8491899

[btab879-B27] Ma Z.S. et al (2019) Diversity-disease relationships and shared species analyses for human microbiome-associated diseases. ISME J., 13, 1911–1919. 30894688 10.1038/s41396-019-0395-y PMC6775969

[btab879-B28] Nguyen N.-P. et al (2016) A perspective on 16s rRNA operational taxonomic unit clustering using sequence similarity. NPJ Biofilms Microbiomes, 2, 1–8. 28721243 10.1038/npjbiofilms.2016.4 PMC5515256

[btab879-B29] Quinn T.P. et al (2020) Interpretable log contrasts for the classification of health biomarkers: a new approach to balance selection. mSystems, 5, e00230-19. 32265314 10.1128/mSystems.00230-19 PMC7141889

[btab879-B30] Rivera-Pinto J. et al (2018) Balances: a new perspective for microbiome analysis. mSystems, 3, e00053-18. 30035234 10.1128/mSystems.00053-18 PMC6050633

[btab879-B31] Scher J.U. , Abramson S.B. (2011) The microbiome and rheumatoid arthritis. Nat. Rev. Rheumatol., 7, 569–578. 21862983 10.1038/nrrheum.2011.121 PMC3275101

[btab879-B32] Schwager E. , Huttenhower C. (2020). *banocc: Bayesian ANalysis Of Compositional Covariance*. R package version 1.14.0.

[btab879-B33] Schwager E. et al (2017) A Bayesian method for detecting pairwise associations in compositional data. PLoS Comput. Biol., 13, e1005852. 29140991 10.1371/journal.pcbi.1005852 PMC5706738

[btab879-B34] Selvaraju R.R. et al (2017). Grad-cam: Visual explanations from deep networks via gradient-based localization. In: *2017 IEEE International Conference on Computer Vision (ICCV)*, pp. 618–626.

[btab879-B35] Sinha R. et al (2015) The microbiome quality control project: baseline study design and future directions. Genome Biol., 16, 276. 26653756 10.1186/s13059-015-0841-8 PMC4674991

[btab879-B36] Smilkov D. et al (2017) Smoothgrad: removing noise by adding noise. CoRR abs/1706.03825.

[btab879-B37] Song M. et al (2020) Influence of the gut microbiome, diet, and environment on risk of colorectal cancer. Gastroenterology, 158, 322–340. 31586566 10.1053/j.gastro.2019.06.048 PMC6957737

[btab879-B38] Sze M.A. et al (2017) Normalization of the microbiota in patients after treatment for colonic lesions. Microbiome, 5, 1–10. 29145893 10.1186/s40168-017-0366-3 PMC5689185

[btab879-B39] Vacca M. et al (2020) The controversial role of human gut lachnospiraceae. Microorganisms, 8, 573. 32326636 10.3390/microorganisms8040573 PMC7232163

[btab879-B40] Vallianou N. et al (2019) Understanding the role of the gut microbiome and microbial metabolites in obesity and obesity-associated metabolic disorders: current evidence and perspectives. Curr. Obes. Rep., 8, 317–332. 31175629 10.1007/s13679-019-00352-2

[btab879-B41] Wang H. (2012) Bayesian graphical Lasso models and efficient posterior computation. Bayesian Anal., 7, 867–886.

[btab879-B42] Wang T. et al (2021) MOGONET integrates multi-omics data using graph convolutional networks allowing patient classification and biomarker identification. Nat. Commun., 12, 1–13. 34103512 10.1038/s41467-021-23774-w PMC8187432

[btab879-B43] Westfall S. et al (2017) Microbiome, probiotics and neurodegenerative diseases: deciphering the gut brain axis. Cell. Mol. Life Sci., 74, 3769–3787. 28643167 10.1007/s00018-017-2550-9 PMC11107790

[btab879-B44] Yachida S. et al (2019) Metagenomic and metabolomic analyses reveal distinct stage-specific phenotypes of the gut microbiota in colorectal cancer. Nat. Med., 25, 968–976. 31171880 10.1038/s41591-019-0458-7

[btab879-B45] Yan K.K. et al (2017) A comparison of graph-and kernel-based–omics data integration algorithms for classifying complex traits. BMC Bioinformatics, 18, 1–13. 29212468 10.1186/s12859-017-1982-4 PMC6389230

[btab879-B46] Zackular J.P. et al (2014) The human gut microbiome as a screening tool for colorectal cancer. Cancer Prev. Res., 7, 1112–1121. 10.1158/1940-6207.CAPR-14-0129 PMC4221363 25104642

[btab879-B47] Zhang M. et al (2018). An end-to-end deep learning architecture for graph classification. In: Thirty-Second AAAI Conference on Artificial Intelligence. AAAI Press, Palo Alto, California USA

