# Supplementary material for: CACONET: a novel classification framework for microbial correlation networks
Source: Bioinformatics. 2022 Jan 4;38(6):1639–47. doi: 10.1093/bioinformatics/btab879 (PMC8896646; doi:10.1093/bioinformatics/btab879)
Supplement: btaa879_Supplementary_Data [file btaa879_supplementary_data.pdf]

CACONET: A novel classification framework for microbial correlation networks

Yuanwei Xu, Katrina Nash, Animesh Acharjee, Georgios V. Gkoutos

Supplementary Note

BAnOCC parameters

For simulated data, the prior for $\mathbf{m}$ was $\mathbf{m} \sim \mathcal{N}(\mathbf{0}, 30\mathbf{I})$ with $\mathbf{I}$ being the identity matrix. The hyperprior for the graphical LASSO shrinkage parameter λ was $\lambda \sim \text{Gamma}(0.5, 5)$ with shape = 0.5 and rate = 5. We simulated four Markov chains using the No-U-Turn sampler, with 5000 iterations per chain and 2500 warm-up iterations. Posterior draws were collected every 20 iterations. The same setting was used for all simulated scenarios listed in the main manuscript.

For MBQC data, $\mathbf{m} \sim \mathcal{N}(\mathbf{0}, 30\mathbf{I})$, $\lambda \sim \text{Gamma}(0.5, 3)$. Four Markov chains were run with 8000 iterations per chain and 4000 warm-up iterations. Posterior draws were collected every 20 iterations. The same setting was used for both Healthy and CRC case groups.

DGCNN hyperparameters and node importance calculation

For all simulated scenarios, we used 4 graph convolution layers with number of output channels [32, 32, 32, 1] and hyperbolic tangent (`tanh`) activation function. No truncation was applied to the concatenated output tensor of the graph convolution layers; in other words, there was no "pooling" step in the SortPooling layer. This is because all input graphs to DGCNN have the same set of nodes. The SortPooling layer was followed by a traditional Conv1D layer with 16 filters, filter size and strides both equal 97, the sum of output channels of graph convolution layers. This was then followed by a MaxPooling with pool size 2, another Conv1D with 32 filters, filter size 5 and stride 1, a Dense layer with 128 neurons and ReLU activation, a dropout layer with rate 0.5, and a final softmax layer. We used Adam optimizer with learning rate 0.0001 and binary cross-entropy loss. We used 50% of "diseased" graphs for n-node importance calculation per run, case and sample size, with n up to 10.

For MBQC data, the same DGCNN architecture and hyperparameters were used. We performed 10 DGCNN runs with 100 epochs per run. We used 30% of CRC graphs for n-node importance calculation per run, with n up to 21.

Supplementary Figures

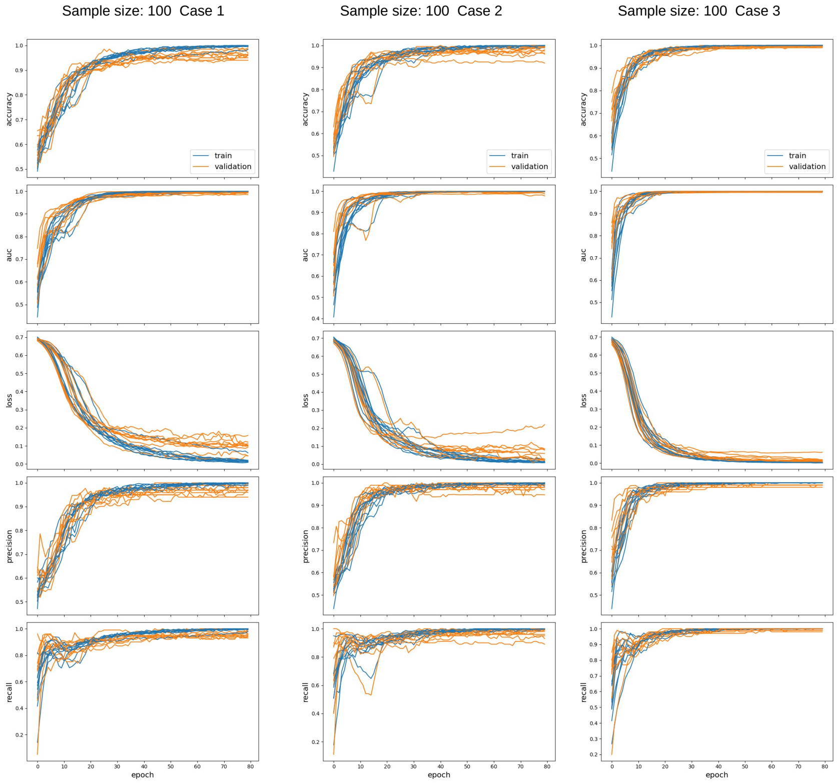

Figure 1: Performance metrics of DGCNN for training and validation data as a function of epoch number, for all cases of sample size 100. Top to bottom: accuracy, AUC, loss, precision, recall.

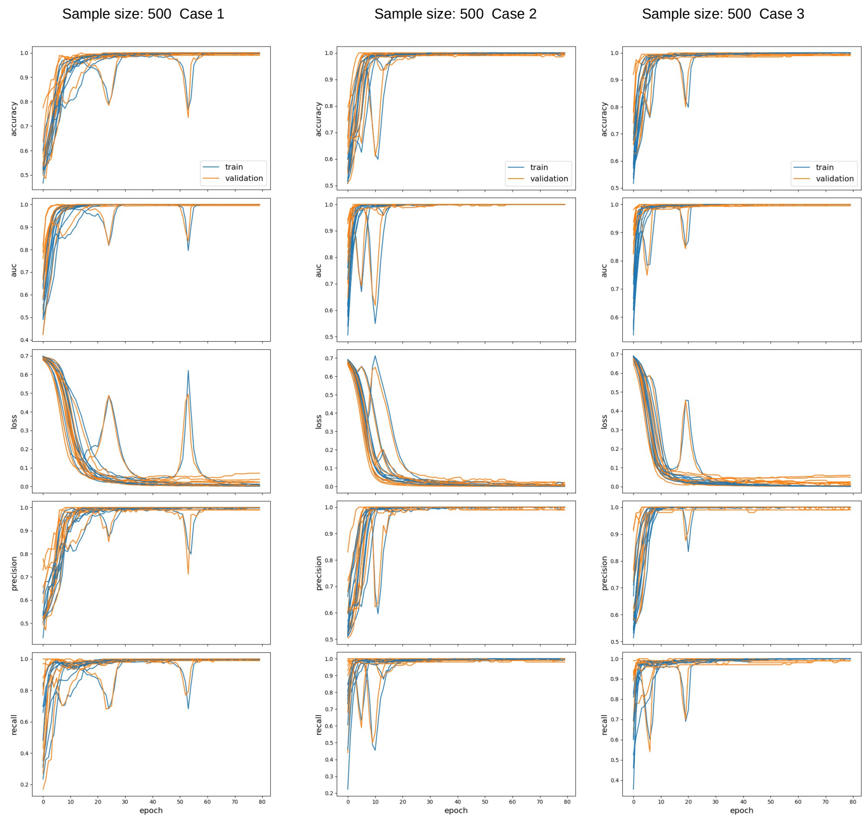

Figure 2: Performance metrics of DGCNN for training and validation data as a function of epoch number, for all cases of sample size 500. Top to bottom: accuracy, AUC, loss, precision, recall.

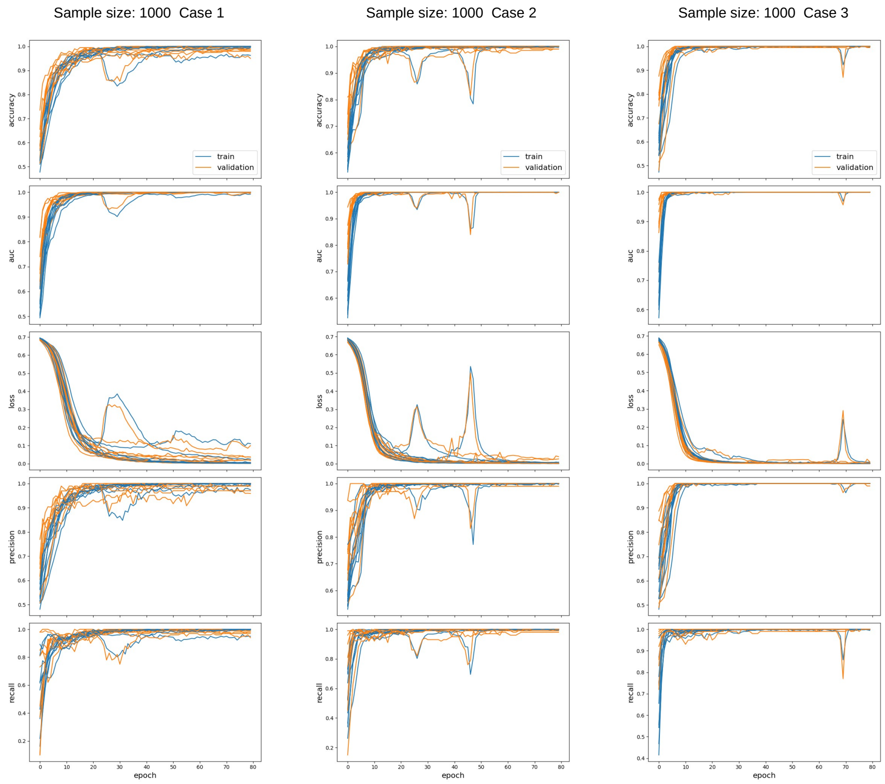

Figure 3: Performance metrics of DGCNN for training and validation data as a function of epoch number, for all cases of sample size 1000. Top to bottom: accuracy, AUC, loss, precision, recall.

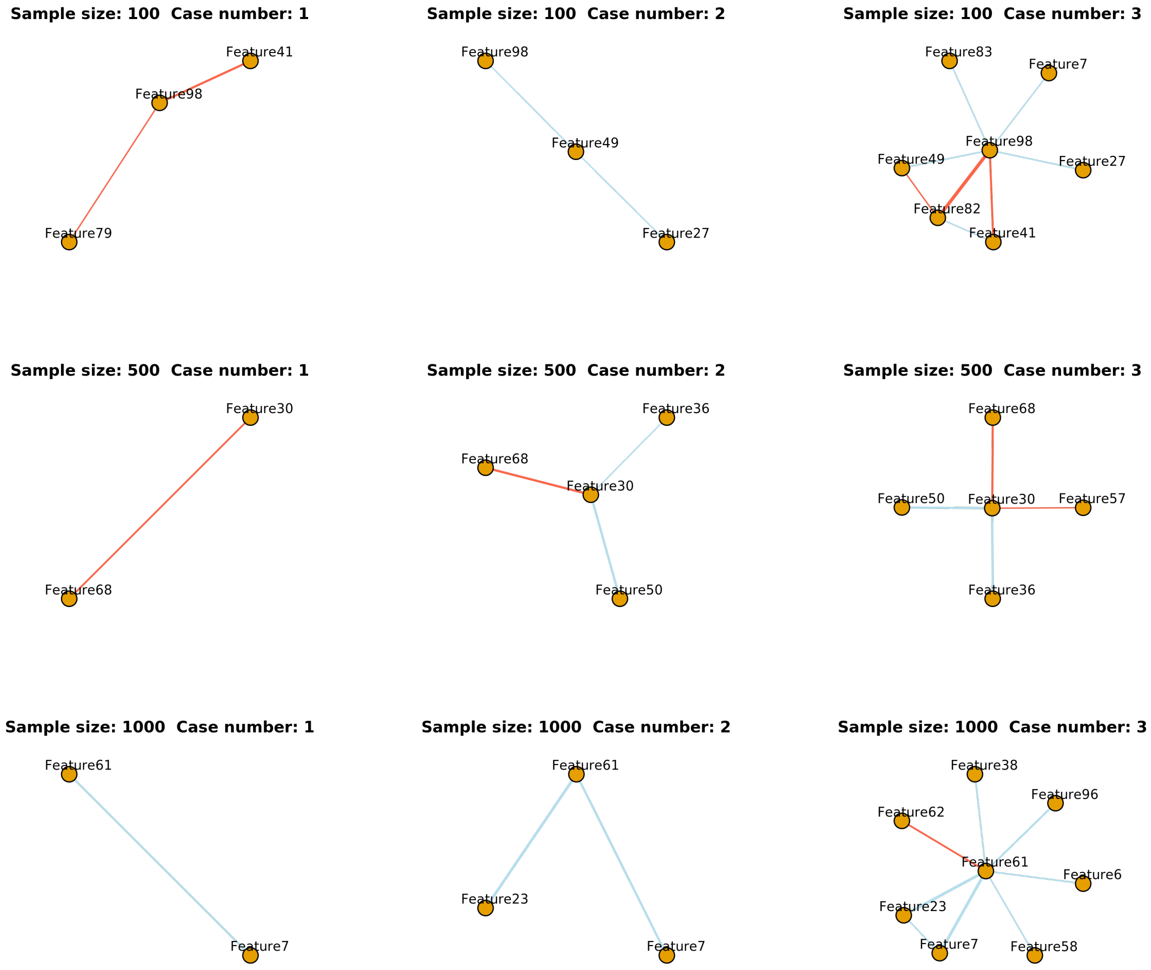

Figure 4: Reconstructed correlation networks from BAnOCC, for all simulated sample sizes and cases listed in the main manuscript. Feature pairs with median correlation strength greater than 0.2 were shown, with edge width proportional to the correlation strength. Positive correlations are shown in blue and negative ones in red.

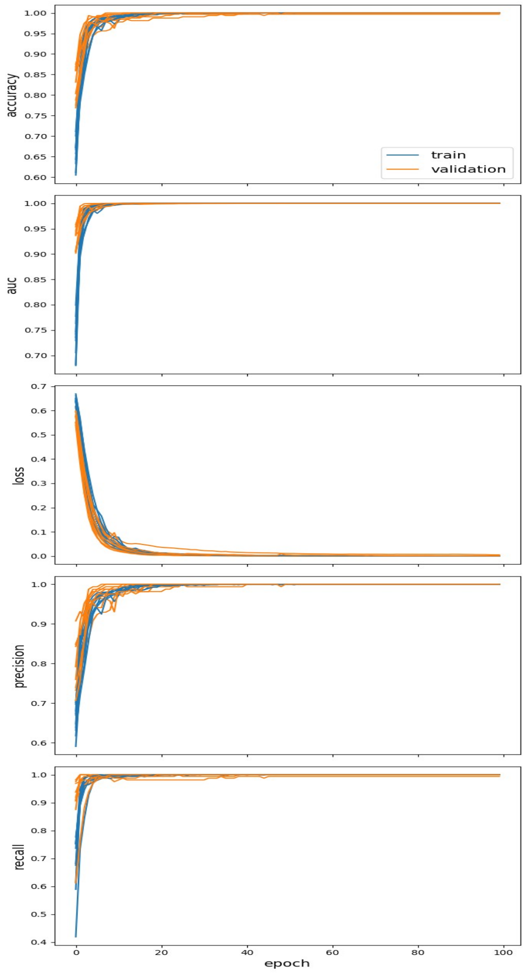

Figure 5: Performance metrics of DGCNN for training and validation data as a function of epoch number, for correlation networks inferred from the OTU table of CRC case and Healthy, as part of the MBQC baseline data. Top to bottom: accuracy, AUC, loss, precision, recall.

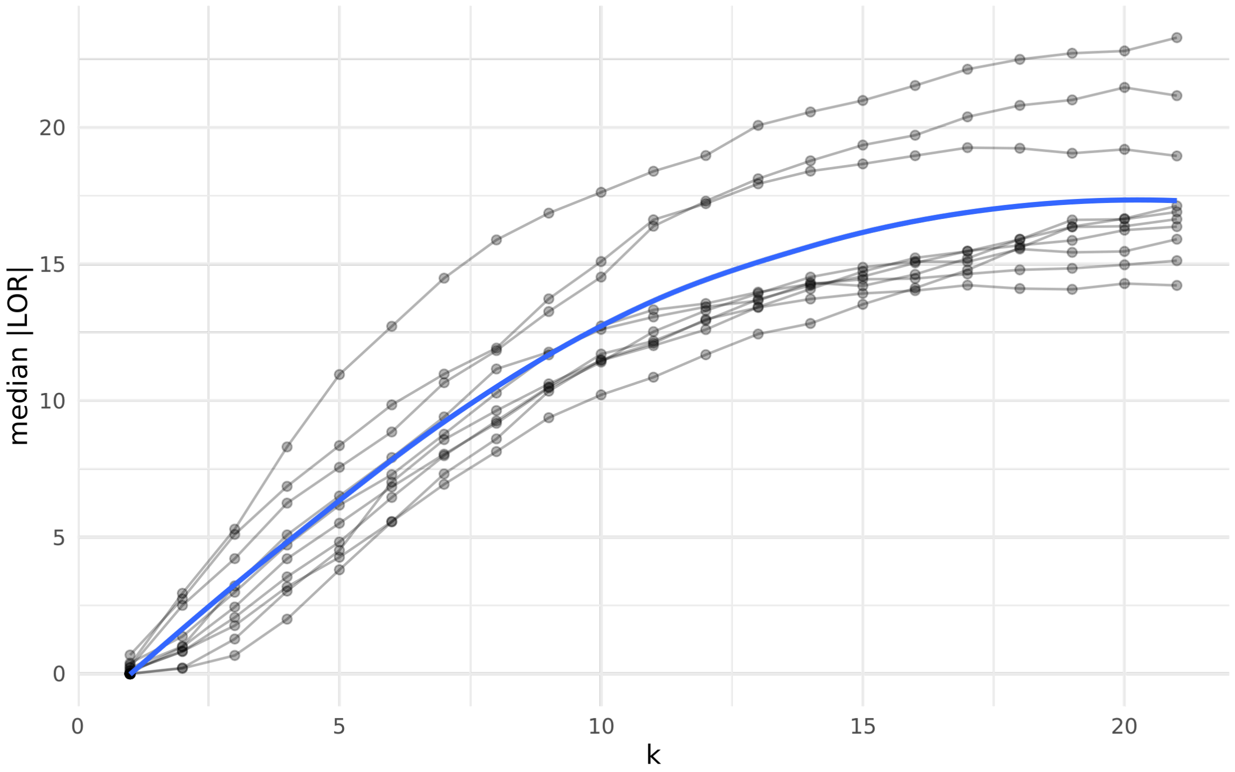

Figure 6: Median absolute LOR as a function of number of knocked-out nodes (k). For each k, the k most important nodes were found by greedy search, and the corresponding LOR was plotted for each DGCNN run. A smoothed line (blue) was fitted to represent the trend of LOR.
